# Clinical and pathological characteristics of NELL-1-positive membranous nephropathy: a case series study

**DOI:** 10.3389/fmed.2025.1615855

**Published:** 2025-09-04

**Authors:** Kang Li, Zheng Liang, Xiaojing Liu, Shuyi Cao

**Affiliations:** Department of Nephrology, Cangzhou Central Hospital, Cangzhou, China

**Keywords:** NELL-1, membranous nephropathy, IgG subclasses, malignancy, case series

## Abstract

**Objective:**

This study aimed to comprehensively characterize the clinical and pathological features, treatment strategies, and outcomes of patients with neural epidermal growth factor-like 1 protein (NELL-1)-positive membranous nephropathy (MN).

**Methods:**

We retrospectively analyzed non-systemic lupus erythematosus (SLE) MN patients diagnosed between January 2010 and August 2021 at Cangzhou Central Hospital, China. Inclusion required negative PLA2R and THSD7A staining and available paraffin-embedded renal tissue. Clinical, laboratory, pathological, treatment, and outcome data were collected and analyzed descriptively.

**Results:**

Among 531 non-SLE MN cases screened, 23 patients (13 males, 10 females; mean age 49.4 ± 12.0 years) were identified as NELL-1(+), PLA2R(−), and THSD7A(−). Hypertension was present in 26.1%, and nephrotic syndrome in 60.9% of patients. Bright granular NELL-1 deposits along glomerular capillary loops were observed in all cases, with segmental or incomplete global distribution seen in 65.2%. IgG4 and IgG1 were the predominant immunoglobulin subclasses (82.6 and 78.2%, respectively). Over a median follow-up of 56 months, 73.9% received immunosuppressive therapy, while 26.1% were managed with supportive treatment alone. The overall remission rate was 87.0% (73.9% complete, 13.0% partial), with only two patients experiencing transient renal function decline and none progressing to end-stage renal disease. Notably, 26.1% of patients developed malignancies, most commonly lung cancer. In patients with malignancy, tumor resection and supportive therapy alone frequently led to remission of MN.

**Conclusion:**

NELL-1-positive MN is characterized by high rates of IgG1 and IgG4 deposition and generally favorable responses to therapy. A substantial proportion of patients were diagnosed with malignancy, highlighting the importance of cancer screening in this population. Timely identification and management of underlying malignancy may contribute to improved renal outcomes.

## Introduction

Membranous nephropathy (MN) is an immune-mediated glomerular disease characterized by immune complexes deposition along the glomerular basement membrane (GBM) and a leading cause of nephrotic syndrome in adults (1, 2). Approximately 80% of cases are primary, while the remaining are associated with autoimmune diseases, infections, drugs, or malignancies (3). The clinical course of MN is highly heterogeneous; some patients experience spontaneous remission, while others progress to end-stage renal disease (ESRD) (4). Studies have demonstrated that the detection of antibodies against podocyte antigens plays a crucial role in the diagnosis and treatment of MN (5). About 70% of MN cases are associated with the phospholipase A2 receptor (PLA2R), and the specificity of anti-PLA2R antibodies for pathological diagnosis reaches up to 99% (6). Additionally, changes in anti-PLA2R antibody titers correlate with treatment responses (7). Thus, anti-PLA2R antibody detection has become an essential tool for the diagnosis and therapeutic management of PLA2R (+) MN. Thrombospondin type-1 domain-containing 7A (THSD7A) protein is another target antigen that is present in 1–5% of primary MN cases (8, 9).

Apart from PLA2R and THSD7A, in 2020, Sethi et al. identified Neural epidermal growth factor-like 1 protein (NELL-1) as another pathogenic antigen in primary membranous nephropathy (MN) (10). NELL-1 is a secreted protein predominantly expressed in neural tissues, with low expression levels in non-neural tissues such as the liver and kidney (11). In normal renal tissue, NELL-1 is more highly expressed in renal tubules and is scarcely detectable in glomeruli (12). Studies of human embryonic kidney cells cultured on gelatin-coated coverslips have shown that NELL-1 deposits in the extracellular matrix, suggesting that it may be an extracellular component capable of depositing in the glomerular basement membrane (GBM) (13).

Recent research has revealed that in PLA2R (−) MN, approximately 16–35% of renal tissues exhibit NELL-1 positivity on immunostaining (10, 14). The deposition pattern of NELL-1 is similar to that of PLA2R, with granular deposition along the capillary walls, consistent with the location of IgG deposits, and no mesangial or subendothelial deposition. This suggests that NELL-1 may be involved in the pathogenesis of MN (12). As a novel biomarker, several studies found that NELL-1 (+) could be associated with Mercury-Related MN (15, 16), while another report showed that NELL-1 (+) was found in 29% of patients with isolated segmental membranous glomerulopathy (17). However, the association between NELL-1 (+) and tumor remained controversial, Wang et al. reported no tumor was found among 15 NELL-1 (+) NM patients (14) while Caza et al. found that about one third of NELL-1(+) patients had a high prevalence of malignant tumors (30/91) (18). However, these studies all had a limited sample size, and further reports on the characteristics of NELL-1 (+) MN patients are still lacking.

Therefore, this study aimed to investigate the clinical and pathological characteristics, treatments, comorbidity, and outcomes of patients with NELL-1 (+) MN.

## Materials and methods

### Study design and patients

This retrospective case series study included non-systemic lupus erythematosus (SLE) MN patients who were hospitalized in the Department of Nephrology at Cangzhou Central Hospital between January 2010 and August 2021 were initially screened. All patients included in the study were Chinese. The Inclusion Criteria were: (1) Patients with renal biopsy pathology revealed diffuse granular IgG deposition on the subepithelial side of the glomerular capillary loops and the diagnosis was consistent with membranous nephropathy (19, 20); (2) patients with negative PLA2R staining in renal tissue and sufficient remaining paraffin-embedded kidney tissue blocks. The exclusion criteria included patients with confirmed autoimmune diseases (e.g., SLE), hepatitis virus infections (e.g., hepatitis B or C), poisoning caused by organic solvents or heavy metals (e.g., mercury poisoning), as well as those with a history of using whitening creams, mercury-containing drugs, or occupational exposure to mercury. This study was conducted in accordance with the Declaration of Helsinki and was approved by the Ethics Committee of Cangzhou Central Hospital, and the requirement of written informed consent was waived by the Ethics Committee due to the retrospectively nature.

### Immunohistochemical staining

Previous paraffin-embedded renal tissue blocks were preserved appropriately in alignment with the guidelines (21) and sectioned to 4 μm thick slices under storage conditions at room temperature away from the melting point of paraffin. Sections were subjected to immunohistochemical staining for NELL-1 (1:800, Sigma-Aldrich) and THSD7A (1:1500 dilution, Sigma-Aldrich). The typical pathological manifestations of NELL-1 were shown in Supplementary Figure 1.

### Data collection

For eligible patients, baseline clinical, laboratory, and pathological data were collected from medical records. Treatment and prognosis data were collected from the medical records or follow-up interviews. Baseline characteristics including age, gender, hypertension status (hypertension was defined as office blood pressure ≥140/90 mmHg) (22), body mass index (BMI), laboratory parameters (including urinary protein, urinary red blood cells, blood biochemistry: albumin, serum creatinine, uric acid, eGFR, cholesterol, triglycerides) of all patients were collected. The pathological features including positivity, intensity, and deposition location of podocyte antigens; and immunological parameters (anti-PLA2R and anti-THSD7A antibodies) were also documented. Mesangial proliferation, glomerulosclerosis, interstitial fibrosis, and tubular atrophy (IFTA) were evaluated under light microscopy and further graded as mild (<25%), moderate (25–50%), or severe (>50%) (23). Immunofluorescence: Assessment of staining intensity and proportion for IgG, IgA, IgM, C3, C4, C1q, *κ*, *λ*, PLA2R, and THSD7A. Determination of the deposition, location proportion of electron-dense deposits, and the proportion of different MN stages were performed using electron microscopy.

Treatment regimens were determined according to KDIGO guidelines and physician assessments, non-immunosuppressive antiproteinuric therapy (NIAT) [such as ACE inhibitors (ACEIs), or angiotensin receptor blockers (ARBs)], and immunosuppressive therapy [such as prednisone, cyclophosphamide (CTX), calcineurin inhibitors (CNIs), cyclosporin A (CsA), tacrolimus (Tac), rituximab (RTX)]. The time from disease onset to tumor diagnosis or progression to ESRD was recorded. Clinical and laboratory parameters were also documented during follow-up, including blood pressure, urinary protein, serum creatinine, eGFR, remission status (complete, partial, or spontaneous), time to remission, occurrence and timing of thrombosis, tumor development, and time to tumor diagnosis.

According to the 2012 KDIGO guidelines (24), complete remission (CR) is defined as: proteinuria < 0.3 g/d and serum albumin > 35 g/L. Partial remission (PR) is defined as: a reduction in proteinuria > 50%, with proteinuria between 0.3 and 3.5 g/d and serum albumin > 30 g/L. Spontaneous remission is defined as achieving CR or PR without the use of immunosuppressive drugs. Transient renal function decline was defined as a reduction in eGFR (estimated glomerular filtration rate) of more than 40% (25), calculated using the CKD-EPI (Chronic Kidney Disease Epidemiology Collaboration) equation, that subsequently recovered to within 10% of the baseline value within 3 months and did not progress to end-stage renal disease (ESRD).

### Statistical analysis

Statistical analysis was performed using SPSS 21.0 (IBM Corp., Armonk, NY, United States), and only descriptive statistics were conducted. Normally distributed continuous variables were expressed as mean ± standard deviation. Non-normally distributed variables were expressed as medians (interquartile range). Categorical variables were expressed as rates (percentages).

## Results

### General characteristics

**Figure 1:**
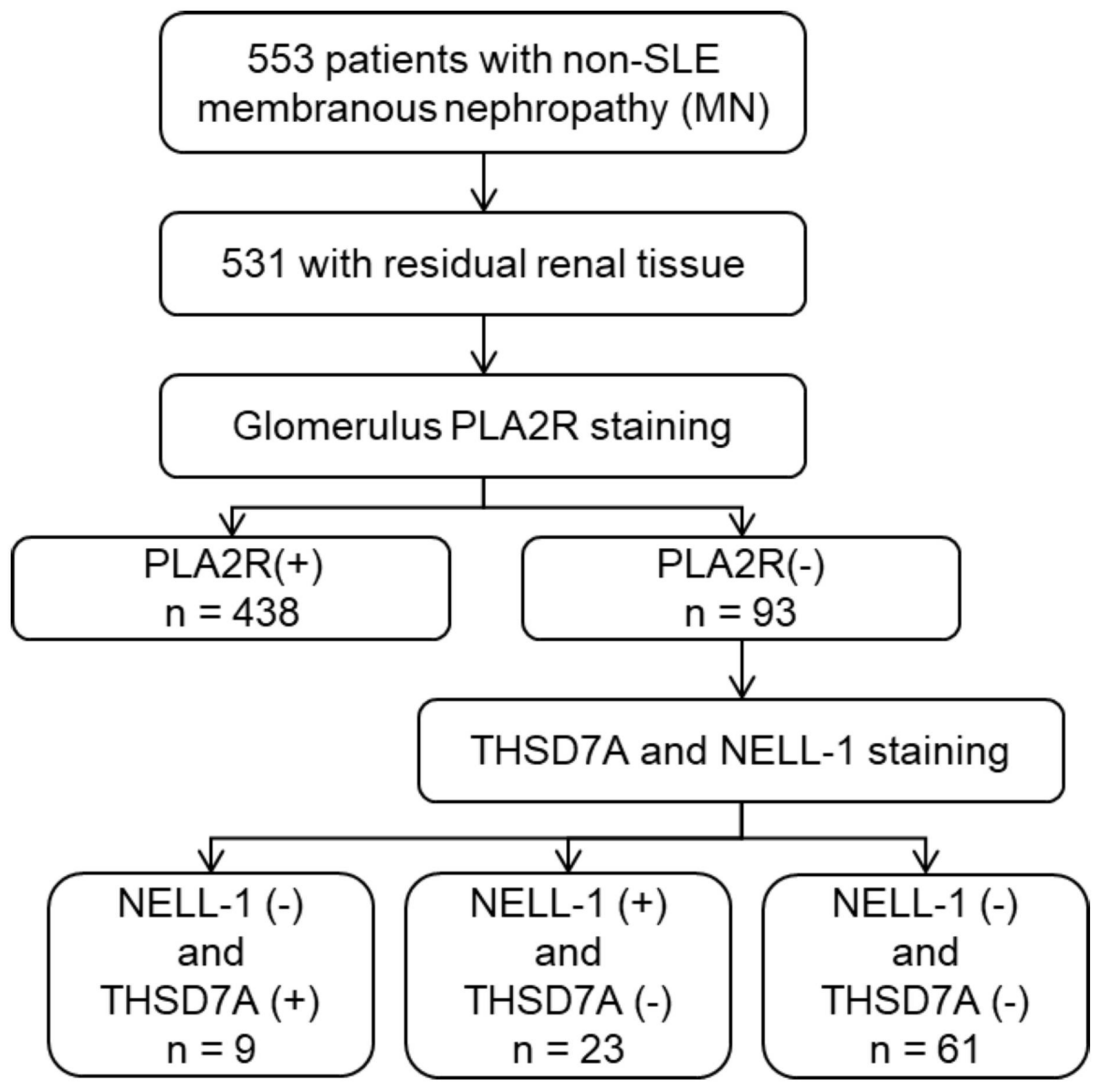
Flowchart of patients.

Between January 2010 and August 2021, a total of 553 patients with non-SLE membranous nephropathy (MN) were initially screened. Of these, 531 had available residual paraffin-embedded renal tissue blocks suitable for further analysis. Among this cohort, 93 patients (17.5%) were negative for PLA2R staining. Twenty-three patients with NELL-1 positivity and negative for both PLA2R and THSD7A were identified and included in the present analysis (Figure 1). The clinical and pathological characteristics of these 23 NELL-1-positive MN patients are summarized in Table 1 and detailed in Supplementary Tables 1, 2. The mean age was 49.4 ± 12.0 years, with a male predominance (56.5%). Hypertension was present in 26.1% of patients. At the time of renal biopsy, the mean eGFR was 113.4 ± 16.1 mL/min/1.73 m^2^, with two patients exhibiting an eGFR <90 mL/min/1.73 m^2^. Nephrotic syndrome was observed in 60.9% of cases.

**Table 1:** Clinical and pathological characteristics.

Characteristics	NELL-1(+) (*n* = 23)
Age (years)	49.35 ± 11.97
Gender, female, n (%)	10(43.5%)
Hypertension, n(%)	6(26.1%)
Proteinuria (g/d)	4.163 ± 1.558
Urinary red blood cells (cells/μL)	8(1–16)
Albumin (g/L)	27.18 ± 5.516
Serum creatinine (μmol/L)	58.83 ± 11.02
eGFR (mL/min/1.73 m^2^)	113.4 ± 16.11
Total cholesterol (mmol/L)	6.927 ± 1.54
Triglycerides (mmol/L)	2.03(1.44–3.11)
Thrombosis, n(%)	0(0.0%)
IFTA	
0	5(21.7%)
1	13(56.6%)
2	5(21.7%)
3	0(0.0%)
Segmental sclerosis, n(%)	3(13.0%)
Mesangial proliferation, n(%)	5(21.7%)
IgG1 positive, n(%)	18(78.2%)
IgG2 positive, n(%)	5(21.7%)
IgG3 positive, n(%)	1(4.3%)
IgG4 positive, n(%)	19(82.6%)
IgA positive, n(%)	3(13%)
Predominantly IgG4 positive, n(%)	13(56.5%)
C1q positive, n(%)	3(13.0%)

Pathological examination revealed segmental sclerosis in 13.0% of patients and mesangial proliferation in 21.7%. The majority of cases showed mild to moderate interstitial fibrosis and tubular atrophy (IFTA). NELL-1 immunostaining demonstrated bright granular deposits along the glomerular capillary loops in all cases, with segmental or incomplete global distribution observed in 30.4 and 34.8% of patients, respectively. The examples of NELL-1 segmental distribution were displayed in Supplementary Figure 1. No cases showed evidence of tubular basement membrane (TBM) deposits. Immunofluorescence analysis revealed that IgG4 and IgG1 were the predominant subclasses among NELL-1-positive MN, detected in 82.6 and 78.2% of patients, respectively. IgG2 and IgG3 positivity was less common. Thirteen patients (56.5%) showed predominantly IgG4-positive staining. None of the cases were dual-positive for both PLA2R and NELL-1. Additionally, none of the patients had clinical histories of conditions previously associated with NELL-1 MN, such as graft-versus-host disease, NSAID use, rheumatoid arthritis, sarcoidosis, or exposure to thiol-containing medications.

### Treatments and outcomes

Seventeen (73.9%) patients received immunosuppressive therapy, while six patients received only ACEIs or ARBs. The 23 patients had a median follow-up of 56 months (range: 48–59 months). During follow-up, 3 patients achieved partial remission (13.0%), 17 achieved complete remission (73.9%), and 6 experienced spontaneous remission. Two patients experienced relapse during follow-up but both achieved remission again after additional immunosuppressive therapy or a change in immunosuppressive drugs. Renal function decline was rare, observed in only two patients (one for 12 months and one for 16 months), and no patients developed end-stage renal disease (ESRD) (Table 2; Supplementary Table 3).

**Table 2:** Treatments and outcomes.

Treatments and outcomes	NELL-1+
NIAT, n(%)	22(95.7%)
P + CNI, n(%)	14(60.9%)
P + CTX, n(%)	4(17.4%)
Follow-up duration (months)	56(48–59)
Remission, n (%)	20(87.0%)
CR, n(%)	17(73.9%)
PR, n(%)	3(13.0%)
SR, n(%)	6(26.1%)
Decline in renal function, n (%)	2(8.7%)
Tumor, n (%)	6(26.1%)

### Malignancy-associated cases

Six patients (26.1%) were diagnosed with malignancies: three with lung carcinoma, one with thyroid carcinoma, one with colorectal carcinoma, and one with prostate carcinoma. The timing of cancer diagnosis, oncologic treatments, and relationship to MN diagnosis and therapy are detailed below:

Case 10: Lung carcinoma was identified concomitantly with MN diagnosis. The patient underwent lung resection and received oral osimertinib for 1 year, achieving complete response (CR) of the cancer. MN was managed with ACEI only, and renal CR was observed 8 months later.
Case 12: Lung carcinoma and MN were diagnosed at the same time. The patient received lung resection without adjuvant therapy and achieved CR for the malignancy. Due to marked edema, immunosuppressive therapy (prednisone plus tacrolimus) was started, and renal CR was achieved after 8 months.
Case 13: Thyroid carcinoma was discovered at two-month follow-up after MN diagnosis. Surgical resection alone led to CR of the malignancy. MN was managed with ARB only, and renal CR was achieved at 7 months.
Case 16: Colorectal carcinoma was detected 1 month after MN diagnosis. The patient had surgical resection only, achieving CR. MN was managed with ACEI, and renal CR was achieved after 8 months.
Case 17: Lung carcinoma was identified at the time of MN diagnosis. The patient underwent resection without adjuvant therapy, achieving CR. Immunosuppressive therapy (prednisone plus tacrolimus) was required due to nephrotic syndrome, and renal CR was achieved after 9 months.
Case 18: Prostate carcinoma was diagnosed 2 months after MN diagnosis. The patient underwent prostatectomy and radiotherapy, resulting in CR of the malignancy. MN was treated with ACEI, and renal CR was achieved at 12 months.

Of these six patients, four received only ACEI or ARB support in addition to cancer treatment, and all achieved complete remission of nephropathy. No tumor recurrence or renal relapse was observed during follow-up. For patients who underwent surgery for their malignancy, in cases where only supportive therapy (ACEI/ARB) was given, complete remission of MN was achieved following tumor resection. This suggests that tumor removal may have contributed to MN resolution in selected patients. In other cases, immunosuppression was required due to severe nephrotic syndrome at onset.

## Discussion

This case series identified 23 patients with NELL-1 (+), PLA2R (−), and THSD7A (−) membranous nephropathy (MN) from a cohort of 531 non-SLE MN patients. IgG4 (+) and IgG1 (+) were the dominant subclasses among these patients, who also demonstrated a relatively good prognosis: during a median follow-up of 56 months, 20 (87.0%) achieved complete or partial remission, only 2 (8.7%) experienced transient renal function decline, and no patient developed end-stage renal disease.

The prevalence of NELL-1 (+) MN among PLA2R (−) THSD7A (−) MN patients in our study was 24.7% (23 out of 93 cases). This finding aligns with a prevalence study by Sethi et al., who reported a 17.1% NELL-1(+) rate among PLA2R(−) patients (10), but is lower than that observed in the study by Wang et al., who reported a 35% positive rate in PLA2R(−) and THSD7A(−) MN (14). In the latter study, most patients were female, whereas in our study, the female-to-male ratio was 1:1.3. Therefore, the potential factors contributing to this gender disparity and their significance in NELL-1 (+) MN require further investigation in larger cohorts.

Consistent with prior reports, IgG1 and IgG4 were the predominant subclasses deposited along the glomerular basement membrane (GBM) in primary MN (26, 27). PLA2R and THSD7A typically co-localize with IgG4 (8, 28). If IgG1, IgG2, and IgG3 are positive but IgG4 is negative or weakly positive, secondary causes should be considered (26, 29). Previous studies found that IgG1 positivity is high in NELL-1 (+) MN, with lower IgG4 positivity. For example, Wang et al. reported only 67% IgG4 dominance, but 73% IgG1 positivity (14). Tiffany Caza et al. found IgG1 staining in all NELL-1 MN cases, with 73.1% IgG1 dominance and 53.7% concurrent IgG4 staining (18). In our cohort, 56.5% had IgG4 dominance, while 78.2% had positive IgG1 staining, and 21.7% showed IgG2 positivity, but IgG4 was still predominant in these cases. These findings highlight the heterogeneity of IgG subclass deposition in NELL-1 (+) MN, and suggest further assessment of IgG subclass expression and exclusion of secondary causes is needed.

Notably, we observed that NELL-1 deposition in our cohort was consistently bright and granular along the glomerular capillary loops, with a segmental or incomplete global distribution in a significant proportion of cases (30.4 and 34.8%, respectively). This observation is consistent with previous studies, such as those by Sethi et al. and Caza et al., which also reported that NELL-1 immunostaining can present as either diffuse or segmental patterns along the capillary walls (10, 18). However, the relatively high proportion of segmental or incomplete global staining in our cohort may suggest greater heterogeneity of antigen distribution in NELL-1-associated MN among Chinese patients. The underlying mechanisms for this pattern remain unclear but may relate to differences in antigen expression, local immune response, or the stage of disease at the time of biopsy. Alternatively, technical factors in tissue processing and staining could contribute to the variability observed. Further studies with larger cohorts and standardized protocols are warranted to clarify the significance and clinical implications of these distinct NELL-1 staining patterns.

The association between NELL-1 (+) MN and malignancy remains controversial, with reported cancer rates varying widely. Sethi et al. reported tumors in 4 of 5 NELL-1(+) patients in their validation cohort (10), while no tumors were detected in their discovery or pilot cohorts. Tiffany Caza et al. found a 33% malignancy rate among NELL-1(+) patients (30/91), compared to 4.2% in PLA2R(+) and 10.8% in THSD7A(+) (18). By contrast, Wang et al. found no malignancies in 15 NELL-1(+) MN patients at diagnosis or after a follow-up of 25 ± 21 months in 12 NELL-1(+) MN patients (14). In our study, 6 of 23 NELL-1(+) MN patients (26.1%) were diagnosed with malignancies, either at the time of MN diagnosis or during follow-up. Importantly, four of these patients achieved remission of MN after tumor resection and supportive therapy alone, suggesting a possible causal link between malignancy and NELL-1 (+) MN in selected cases. This finding underscores the importance of thorough malignancy screening in patients with NELL-1 (+) MN.

To date, long-term outcomes and treatment responses of NELL-1 (+) MN patients, compared with NELL-1 (−) MN, remain unclear. To our knowledge, no study has performed long-term follow-up in a NELL-1 (+) MN population. Some case reports described significant renal improvement after supportive care (30) or modified Ponticelli regimen (16), and spontaneous remission after removal of NELL-1 (+) colon carcinoma (31). Collectively, these findings indicate that NELL-1 could be an important biomarker for MN treatment decisions and cancer surveillance, warranting further study.

Despite these strengths, this study is limited by its retrospective design and relatively small sample size, which may restrict the generalizability of our findings. Additionally, due to the low NELL-1 (+) rate and the limited number of NELL-1 (+) patients, we were unable to conduct a comparative analysis with NELL-1 (−) MN. Although the median follow-up was 56 months, no endpoint events (death or progression to ESRD) were observed, precluding formal survival analysis. Future studies should include larger, prospective cohorts with extended follow-up to clarify these associations and outcomes.

## Conclusion

In conclusion, patients with NELL-1 (+) MN in our cohort exhibited a high frequency of IgG1 (+) and IgG4 (+) glomerular deposition and generally favorable treatment responses. Most patients received immunosuppressive therapy, and the overall prognosis was good. Importantly, our results highlight a notable association between NELL-1 (+) MN and malignancy, with over a quarter of patients diagnosed with cancer. This finding supports the need for thorough cancer screening in all patients with NELL-1 (+) MN, as prompt identification and management of underlying malignancy may directly influence renal outcomes.

## Data Availability

The original contributions presented in the study are included in the article/Supplementary material, further inquiries can be directed to the corresponding author.

## Supplementary material

Supplementary Figure 1 NELL-1 immunostaining with segmental distribution. **(A)** 400x magnification. **(B)** 200x magnification.
