# Supplementary figures and images for: Clinical and pathological characteristics of NELL-1-positive membranous nephropathy: a case series study
Source: Front Med (Lausanne). 2025 Sep 4;12:1615855. doi: 10.3389/fmed.2025.1615855 (PMC12443679; doi:10.3389/fmed.2025.1615855)

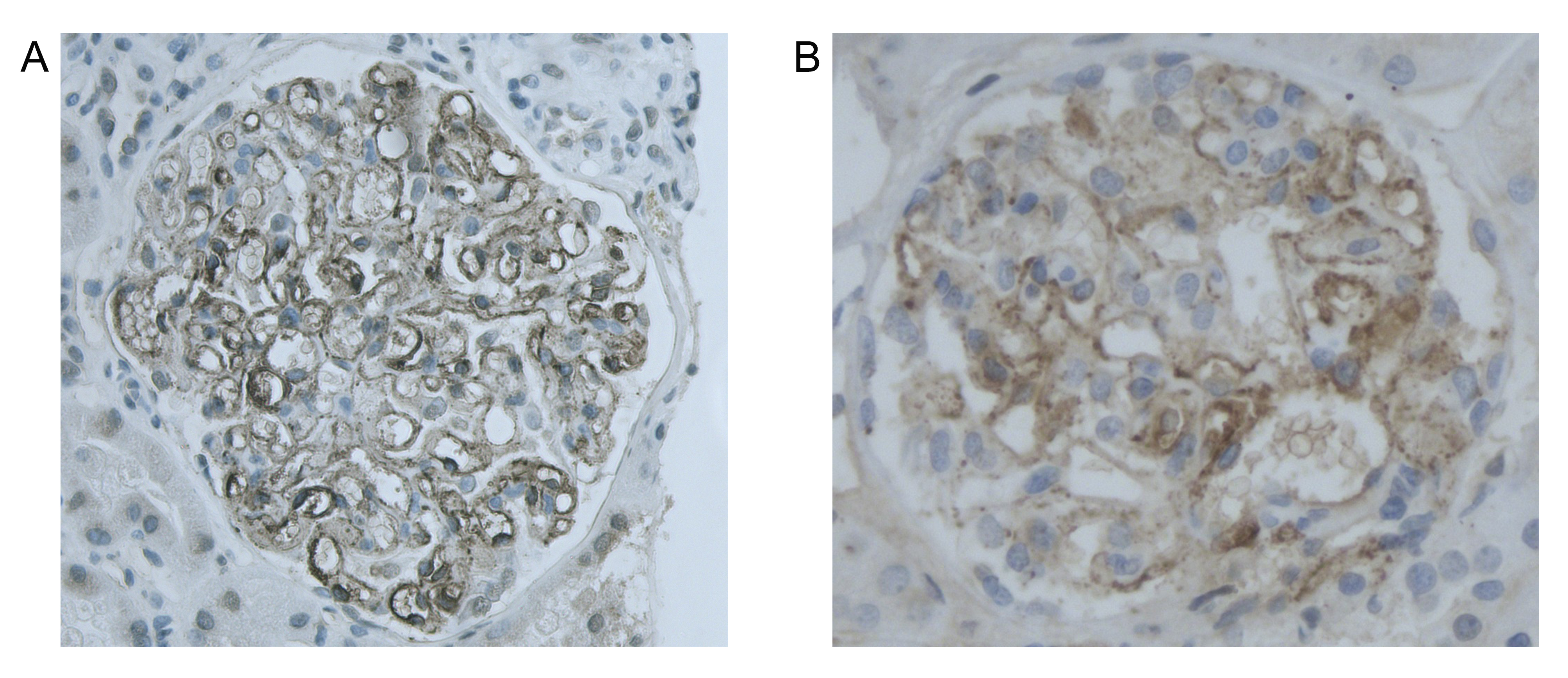

Supplement: Supplementary Figure 1 — NELL-1 immunostaining with segmental distribution. (A) 400x magnification. (B) 200x magnification. [file Image_1.jpeg]
