# Supplementary material for: Clinical and pathological characteristics of NELL-1-positive membranous nephropathy: a case series study
Source: Front Med (Lausanne). 2025 Sep 4;12:1615855. doi: 10.3389/fmed.2025.1615855 (PMC12443679; doi:10.3389/fmed.2025.1615855)
Supplement: Supplementary file 2 [file Table_1.docx]

**Supplementary Table 1. Details of clinical characteristics**

| Case  number | Gender | Age | Hypertension | BMI | Urinary Protein | Proteinuria Range in Kidney Disease | Urinary Red Blood Cells | Hemoglobin | Serum Albumin | Serum Creatinine | eGFR | Uric Acid | Total Cholesterol | Triglycerides |
| --- | --- | --- | --- | --- | --- | --- | --- | --- | --- | --- | --- | --- | --- | --- |
| Case# 1 | Famale | 47 | 0 | 22.96 | 7.05 | 1 | 16 | 127 | 22.2 | 33 | 126.9 | 312 | 7.59 | 5.05 |
| Case# 2 | Male | 67 | 1 | 26.73 | 5.16 | 1 | 7 | 133 | 15.7 | 81 | 86.2 | 456 | 9.27 | 1.17 |
| Case# 3 | Famale | 53 | 0 | 26.03 | 1.04 | 0 | 29 | 139 | 37.5 | 39 | 115 | 308 | 5.42 | 3.11 |
| Case# 4 | Male | 31 | 1 | 32.39 | 3.74 | 1 | 0 | 158 | 29.2 | 55 | 132 | 407 | 5.67 | 2.88 |
| Case# 5 | Male | 57 | 0 | 24.09 | 3.65 | 1 | 2 | 158 | 33.8 | 70 | 99.6 | 313 | 4.77 | 1.44 |
| Case# 6 | Famale | 64 | 0 | 25.3 | 3.65 | 1 | 8 | 125 | 30.1 | 56 | 96 | 255 | 6.36 | 2.02 |
| Case# 7 | Male | 31 | 0 | 25.88 | 4.42 | 1 | 0 | 151 | 29.9 | 67 | 122 | 403 | 5.15 | 4.62 |
| Case# 8 | Male | 56 | 0 | 25.86 | 3.72 | 1 | 30.6 | 113 | 30.6 | 50 | 115.2 | 481 | 4.31 | 0.96 |
| Case# 9 | Famale | 60 | 0 | 26.17 | 3.67 | 1 | 15 | 139 | 20.7 | 65 | 89.4 | 334 | 7.02 | 2.19 |
| Case# 10 | Famale | 32 | 0 | 20.81 | 2.99 | 0 | 10 | 117 | 31.2 | 54 | 119.9 | 270 | 5.57 | 1.05 |
| Case# 11 | Male | 35 | 0 | 27.13 | 6.22 | 1 | 0 | 142 | 25.8 | 71 | 115.6 | 440 | 8.32 | 1.85 |
| Case# 12 | Famale | 51 | 0 | 25.39 | 4.07 | 1 | 0 | 136 | 26.7 | 47 | 110.6 | 432 | 7.32 | 2.03 |
| Case# 13 | Famale | 65 | 1 | 25.8 | 2.62 | 0 | 0 | 116 | 32.9 | 55 | 94.5 | 338 | 8.45 | 2.1 |
| Case# 14 | Famale | 64 | 0 | 25.3 | 3.65 | 1 | 8 | 125 | 28.1 | 56 | 94.6 | 255 | 6.36 | 2.02 |
| Case# 15 | Male | 51 | 1 | 26.9 | 3.71 | 1 | 8 | 150 | 25.3 | 64 | 128 | 305 | 5.9 | 1.21 |
| Case# 16 | Male | 41 | 0 | 20.44 | 2.46 | 0 | 9 | 130 | 34.6 | 62 | 107.7 | 285 | 5.35 | 1.04 |
| Case# 17 | Male | 51 | 1 | 25.22 | 3.59 | 1 | 5 | 126 | 24.7 | 57 | 147.5 | 411 | 7.8 | 3.11 |
| Case# 18 | Male | 48 | 1 | 33.9 | 2.16 | 0 | 10 | 166 | 34.7 | 68 | 121.7 | 438 | 6.43 | 3.03 |
| Case# 19 | Male | 53 | 0 | 22.03 | 4.64 | 1 | 146 | 134 | 29.2 | 50 | 128.4 | 320 | 9.42 | 5.68 |
| Case# 20 | Male | 47 | 0 | 37.12 | 5.57 | 1 | 12 | 153 | 22 | 59 | 114.6 | 520 | 7.14 | 7.59 |
| Case# 21 | Male | 49 | 0 | 20.76 | 5.34 | 1 | 1 | 148 | 25.1 | 72 | 104.1 | 309 | 7.51 | 1.44 |
| Case# 22 | Male | 25 | 0 | 37.98 | 7.82 | 1 | 27 | 170 | 22.9 | 55 | 137.7 | 516 | 8.76 | 8.57 |
| Case# 23 | Male | 57 | 0 | 25.95 | 4.8 | 1 | 45 | 170 | 19.2 | 67 | 101.4 | 408 | 9.44 | 1.83 |

Note: eGFR was calculated using the CKD-EPI formula.

**Supplementary Table 2. Details of pathological characteristics**

| **Case**  **number** | **NELL-1 Deposition Pattern** | **Glomerulosclerosis** | **Segmental Sclerosis** | **IFTA** | **Presence of Mesangial Proliferation** | **IgG** | **IgG1** | **IgG2** | **IgG3** | **IgG4** | **IgA** | **IgM** | **C3** | **C1q** | **k** | **λ** |
| --- | --- | --- | --- | --- | --- | --- | --- | --- | --- | --- | --- | --- | --- | --- | --- | --- |
| Case# 1 | Segment | 0 | 0 | 1 | 0 | +++ | ++ | + | 0 | +++ | 0 | 0 | ++ | 0 | +++ | +++ |
| Case# 2 | Segment | 1 | 1 | 1 | 0 | +++ | ++ | 0 | 0 | +++ | 0 | 0 | ++ | + | +++ | +++ |
| Case# 3 | Diffuse Glomerular | 0 | 0 | 0 | 0 | +++ | ++ | 0 | 0 | +++ | 0 | 0 | 0 | 0 | +++ | +++ |
| Case# 4 | Incomplete Global | 0 | 0 | 2 | 1 | ++ | ++ | + | 0 | 0 | +-++ | 0 | ++ | 0 | + | + |
| Case# 5 | Incomplete Global | 1 | 0 | 1 | 0 | +++ | ++ | 0 | 0 | +++ | 0 | 0 | ++ | 0 | +++ | +++ |
| Case# 6 | Diffuse Glomerular | 1 | 1 | 2 | 0 | +++ | ++~+++ | 0 | 0 | +++ | 0 | 0 | 0 | 0 | +++ | +++ |
| Case# 7 | Incomplete Global | 0 | 0 | 1 | 0 | +++ | ++ | 0 | 0 | +++ | 0 | 0 | +++ | 0 | +++ | +++ |
| Case# 8 | Diffuse Glomerular | 0 | 0 | 0 | 0 | +++ | ++ | 0 | 0 | +++ | 0 | 0 | ++ | 0 | +++ | +++ |
| Case# 9 | Incomplete Global | 0 | 0 | 2 | 1 | +++ | ++ | 0 | 0 | +++ | 0 | + | ++ | 0 | +++ | +++ |
| Case# 10 | Segment | 0 | 0 | 1 | 1 | +++ | +++ | 0 | 0 | +~++ | + | 0 | ++ | + | +++ | +++ |
| Case# 11 | Segment | 0 | 0 | 1 | 0 | +++ | 0 | + | 0 | +++ | 0 | 0 | ++ | 0 | +++ | +++ |
| Case# 12 | Incomplete Global | 0 | 0 | 1 | 1 | +++ | +++ | 0 | 0 | + | + | 0 | ++ | 0 | +++ | +++ |
| Case# 13 | Diffuse Glomerular | 0 | 0 | 1 | 0 | +++ | ++ | 0 | 0 | +++ | 0 | 0 | +++ | 0 | +++ | +++ |
| Case# 14 | Diffuse Glomerular | 1 | 0 | 2 | 0 | +++ | 0 | + | 0 | +++ | 0 | 0 | 0 | 0 | +++ | +++ |
| Case# 15 | Segment | 0 | 0 | 0 | 0 | +++ | +++ | 0 | 0 | +~++ | 0 | ++ | ++ | + | +++ | +++ |
| Case# 16 | Incomplete Global | 0 | 0 | 0 | 1 | ++ | ++ | 0 | 0 | 0 | 0 | 0 | ++ | 0 | +++ | +++ |
| Case# 17 | Segment | 0 | 0 | 0 | 0 | ++ | 0 | 0 | + | +++ | 0 | 0 | ++ | 0 | ++ | ++ |
| Case# 18 | Segment | 0 | 0 | 1 | 0 | ++ | ++ | 0 | 0 | 0 | 0 | 0 | ++ | 0 | ++ | ++ |
| Case# 19 | Incomplete Global | 0 | 0 | 1 | 0 | ++ | ++ | 0 | 0 | 0 | 0 | 0 | + | 0 | ++ | ++ |
| Case# 20 | Diffuse Glomerular | 1 | 1 | 2 | 0 | +++ | 0 | 0 | 0 | +++ | 0 | 0 | ++ | 0 | +++ | +++ |
| Case# 21 | Diffuse Glomerular | 0 | 0 | 1 | 0 | +++ | 0 | ++ | 0 | +++ | 0 | 0 | ++ | 0 | +++ | +++ |
| Case# 22 | Diffuse Glomerular | 0 | 0 | 1 | 0 | +++ | ++~+++ | 0 | 0 | +++ | 0 | 0 | 0 | 0 | ++ | ++ |
| Case# 23 | Incomplete Global | 0 | 0 | 1 | 0 | +++ | ++ | 0 | 0 | +++ | 0 | 0 | ++ | 0 | + | + |

Note: NELL-1+ deposition along the glomerular capillary loops exhibits an incomplete global distribution: >50%, with segmental distribution <50%.

**Supplementary Table 3. Details of treatments and outcomes**

| Case  number | Induction Therapy Regimen | Maintenance Therapy Regimen | Follow-up Duration | Remission | Spontaneous Remission | Remission Time | Relapse | Number of Relapses | Post-relapse Treatment Regimen | Post-relapse Remission | Thrombosis | Cancer | Cancer Onset Time | Decline in Renal Function | Renal Function Decline Time | End-stage Renal Disease (ESRD |
| --- | --- | --- | --- | --- | --- | --- | --- | --- | --- | --- | --- | --- | --- | --- | --- | --- |
| Case# 1 | P+FK506 | P+FK506 | 48 | CR | 0 | 12 | 0 | - | - | - | 0 | 0 | - | 0 | - | 0 |
| Case# 2 | P+CTX | ARB | 60 | CR | 0 | 6 | 0 | - | - | - | 0 | 0 | - | 0 | - | 0 |
| Case# 3 | ACEI | ACEI | 60 | CR | 1 | 7 | 0 | - | - | - | 0 | 0 | - | 0 | - | 0 |
| Case# 4 | P+FK506 | P+FK506 | 36 | 0 | 0 | 0 | 0 | - | - | - | 0 | 0 | - | 0 | - | 0 |
| Case# 5 | P+CsA | P+CsA | 58 | PR | 0 | 4 | 0 | - | - | - | 0 | 0 | - | 0 | - | 0 |
| Case# 6 | P+FK506 | P+FK506 | 47 | PR | 0 | 6 | 1 | 1 | P+CTX | PR | 0 | 0 | - | 1 | 16 | 0 |
| Case# 7 | ACEI | ACEI | 56 | PR | 1 | 10 | 0 | - | - | - | 0 | 0 | - | 0 | - | 0 |
| Case# 8 | P+CsA | P+CsA | 48 | CR | 0 | 2 | 0 | - | - | - | 0 | 0 | - | 0 | - | 0 |
| Case# 9 | P+CTX | ARB | 56 | CR | 0 | 11 | 0 | - | - | - | 0 | 0 | - | 0 | - | 0 |
| Case# 10 | ACEI | ACEI | 63 | CR | 1 | 8 | 0 | - | - | - | 0 | Lung Cancer | 1 | 0 | - | 0 |
| Case# 11 | P+FK506 | P+FK506 | 59 | CR | 0 | 12 | 1 | 1 | P+FK506 | CR | 0 | 0 | - | 0 | - | 0 |
| Case# 12 | P+FK506 | P+FK506 | 48 | CR | 0 | 8 | 0 | - | - | - | 0 | Lung Cancer | 1 | 0 | - | 0 |
| Case# 13 | ARB | ARB | 45 | CR | 1 | 7 | 0 | - | - | - | 0 | Thyroid Cancer | 1 | 0 | - | 0 |
| Case# 14 | P+CsA | P+CsA | 56 | CR | 0 | 12 | 0 | - | - | - | 0 | 0 | - | 0 | - | 0 |
| Case# 15 | P+FK506 | P+FK506 | 52 | CR | 0 | 5 | 0 | - | - | - | 0 | 0 | - | 0 | - | 0 |
| Case# 16 | ACEI | ACEI | 59 | CR | 1 | 8 | 0 | - | - | - | 0 | Colon Cancer | 3 | 0 | - | 0 |
| Case# 17 | P+FK506 | P+FK506 | 54 | CR | 0 | 9 | 0 | - | - | - | 0 | Lung Cancer | 6 | 0 | - | 0 |
| Case# 18 | ACEI | ACEI | 49 | CR | 1 | 12 | 0 | - | - | - | 0 | Prostate Cancer | 1 | 0 | - | 0 |
| Case# 19 | P+CsA | P+CsA | 56 | CR | 0 | 18 | 0 | - | - | - | 0 | 0 | - | 0 | - | 0 |
| Case# 20 | P+CsA | P+CsA | 58 | CR | 0 | 5 | 0 | - | - | - | 0 | 0 | - | 0 | - | 0 |
| Case# 21 | P+FK506 | P+FK506 | 38 | CR | 0 | 8 | 0 | - | - | - | 0 | 0 | - | 0 | - | 0 |
| Case# 22 | P+CTX | ACEI | 63 | 0 | 0 | 0 | 0 | - | - | - | 0 | 0 | - | 1 | 12 | 0 |
| Case# 23 | P+CTX | ARB | 53 | 0 | 0 | 0 | 0 | - | - | - | 0 | 0 | - | 0 | - | 0 |

Note: P: Prednisone; CsA: Cyclosporin A; CTX: Cyclophosphamide; NIAT: Non-immunosuppressive therapy, including ACEI and ARB, where ACEI: Angiotensin-converting enzyme inhibitors, ARB: Angiotensin II receptor blockers. All patients who received immunosuppressive treatment were concurrently treated with ACEI/ARB. The remission time refers to the period from diagnosis to the achievement of remission.
